# A study on the impact of marital status on the survival status of prostate cancer patients based on propensity score matching

**DOI:** 10.1038/s41598-024-56145-8

**Published:** 2024-03-14

**Authors:** Qingquan Chen, Xi Zhu, Yiming Hu, Yao Chen, Rongrong Dai, Jiaxin Li, Jiajing Zhuang, Yifei Lin, Yifu Zeng, Liuxia You, Yanyu Zeng, Qian Huang

**Affiliations:** 1 The Second Affiliated Hospital of Fujian Medical University, Quanzhou, 362000 Fujian China; 2 The School of Public Health, Fujian Medical University, Fuzhou, 350108 Fujian China; 3 National Center for Chronic and Noncommunicable Disease Control and Prevention, Chinese Center for Disease Control and Prevention, Beijing, 100050 China; 4 Anyang University, Anyang, 455000 Henan China; 5 The School of Clinical Medicine, Fujian Medical University, Fuzhou, 350108 Fujian China; 6 Cyberspace Institute of Advanced Technology, Guangzhou University, Guangzhou, China

**Keywords:** Prostate cancer, SEER database, Propensity score matching, Marital status, Cancer epidemiology, Urological cancer, Public health

## Abstract

Marital status is an independent prognostic factor for survival in many types of cancers, but its prognostic impact on patients with prostate cancer (PCa) has not been established. The aim of this study was to explore the independent prognostic factors of PCa and to investigate the effect of marital status on survival outcomes in patients with different stratified by PCa. Using the surveillance, epidemiology, and end results (SEER) database, we collected data on 584,655 PCa patients diagnosed between 1975 and 2019. Marital status was classified as married, divorced, widowed, and single. We used the Kaplan–Meier analysis and single multivariate Cox proportional hazards regression analysis to determine the effect of marital status on overall survival (OS) and cancer-specific survival (CSS). In addition, we performed subgroup analyses for different ages, Gleason score and PSA values, and performed a 1:1 propensity score matching (PSM) to reduce the impact of confounding factors to obtain more accurate matching results. According to our findings, marital status was an independent prognostic factor for the survival of PCa patients and a better prognosis of married patients. Moreover, we also found that factors such as age, TNM stage, Gleason score, and PSA concentration were also considered as important predictors for the prognosis of PCa. The above findings can facilitate early detection and treatment of high-risk PCa patients, prolong their life and reduce family burden.

## Introduction

Prostate cancer (PCa) is the most prevalent malignancy in the male population, ranking as the third leading cause of cancer-related deaths among men in the United States, following lung and colorectal cancer^1,2^. This disease primarily affects older men, with 10 out of every 16 cases diagnosed in individuals aged 65 or older, and the mean age at the time of diagnosis is approximately 66 years^3^. The recognized risk factors include age, genetic predisposition, family history of prostate cancer, and approximately 270 genetic variants estimated to contribute to familial relative risk^4,5^.

PCa is a multi-factorial complex disease influenced by various environmental, physiological, immunological, and genetic factors^6^. Many factors and risk factors are associated with prostate cancer, such as age, family history, and race, along with dietary factors, among others^7,8^. Moreover, psychological and social factors have gained increasing attention in cancer research in recent years with the emergence of biopsychosocial medical models^9^. It’s noteworthy that marital status has been shown to be an independent predictor associated with improved survival odds in various cancer types^10^. However, its impact on the survival rate of prostate cancer (PCa) patients remains a subject of debate.

According to the definition of “high-risk prostate cancer” by the US national comprehensive cancer network (NCCN), which can be found at [include a citation link], high-risk disease is characterized by T3a, Gleason score ≥ 8, or PSA ≥ 20, while “extremely high risk” includes T3b or T4 disease. High-risk prostate cancer diagnoses account for approximately 15% of all cases and have the potential to develop into a lethal phenotype^2^. The primary objective of this study was to stratify the independent risk factors identified using the surveillance, epidemiological, and end results (SEER) database and investigate the impact of marital status on the survival of PCa patients after stratification.

## Method

### Patient

Prostate cancer (PCa) patients were identified within the SEER-8 Cancer Medical Registry for the specified year using SEER * Stat software (version 8.4.2). The histological code from the third edition of the International Classification of Oncology Diseases (ICD-O-3) was employed to screen for PCa patients, and, based on histological criteria, only those diagnosed with primary malignancy were included. Data were extracted from the SEER database, encompassing 584,655 PCa patients diagnosed with high-risk prostate cancer between 1975 and 2019.

### Study variables

The SEER database encompasses a wide range of variables, including Race (White, Black, and Other categories encompassing American Indian/AK Native and Asian/Pacific Islander), Year of diagnosis, age (ranging from 0 to 85 +), marital status (Married, Single, Unmarried, Widowed, and Unknown), 6th TNM stage (spanning from 2004 to 2015), Grade stage (categorized as Well-differentiated; Grade I, Moderately-differentiated; Grade II, Poorly-differentiated; Grade III, and Undifferentiated; anaplastic; Grade IV), Tumor size, tumor invasion, Gleason score, and PSA levels, among others. Notably, therapeutic classifications such as chemotherapy, surgery, and radiotherapy were derived from SEER-specific records. Surgery data was selected using the SEER code “RX Summ—Surg Prim Site (1998 +),” while Radiation and Chemotherapy were assessed through the “Radiation recode” and “Chemotherapy recode (yes, no/unk)” in SEER. Furthermore, Vital status recode (utilizing the study cutoff), SEER cause-specific death classification, and survival time information were all sourced from the SEER database. Age was reclassified into two groups: < 65 years and ≥ 65 years, with overall survival (OS) results serving as the primary endpoint for this study.

### Inclusion and exclusion

**Figure 1:**
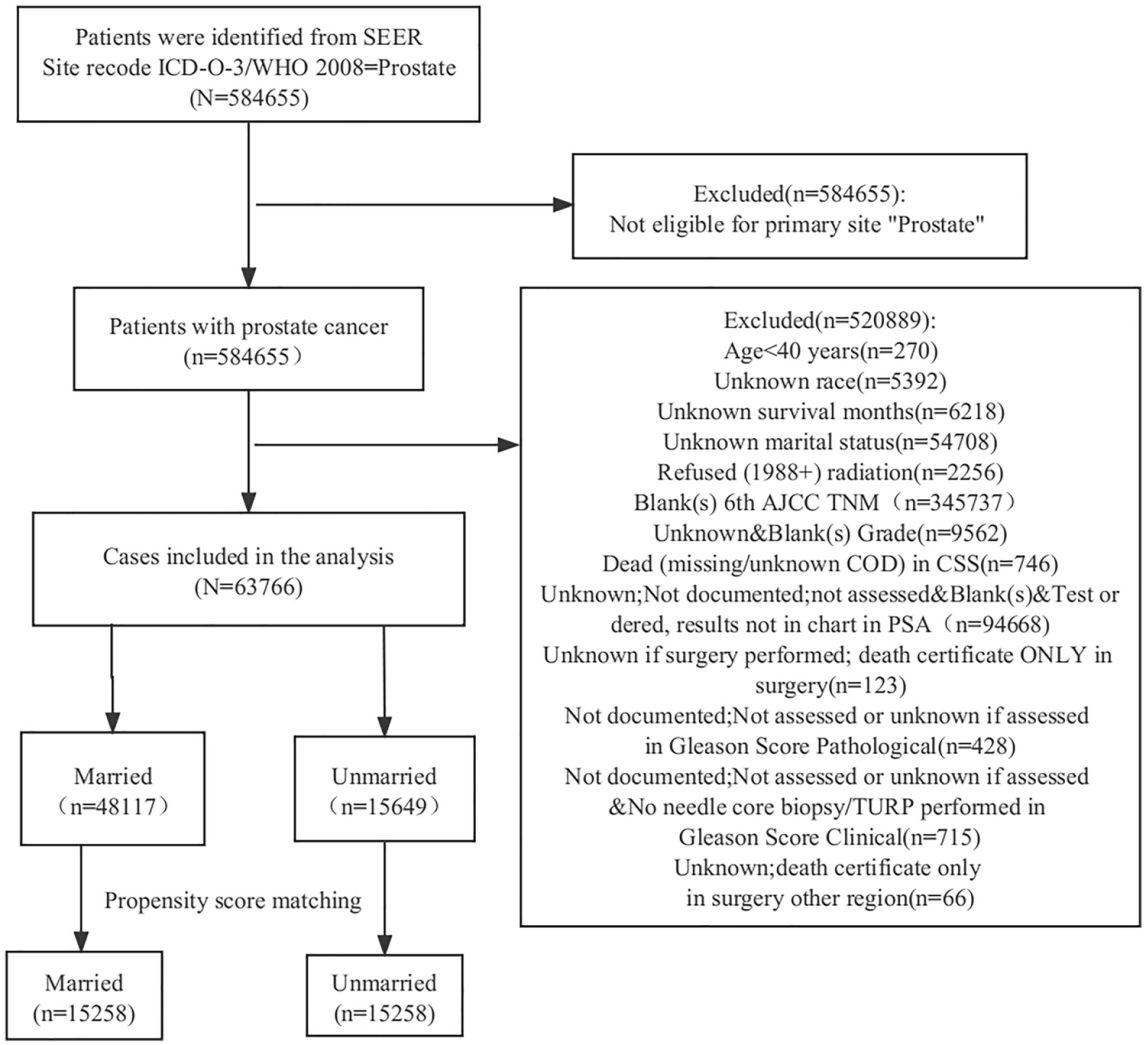
The flow-process diagram for selecting patients based on inclusion and exclusion criteria.

The inclusion criteria for this study were as follows: (1) A confirmed diagnosis of prostate cancer (ICD-O-3); (2) diagnosis between 2004 and 2015 with a minimum follow-up period of 23 years; (3) availability of data regarding survival months and specific causes of death; (4) diagnostic confirmation based on positive histological findings; (5) availability of data on age, marital status, and ethnicity; (6) knowledge of 6th TNM stage; (7) knowledge of PSA values, Gleason score, and tumor grade at the time of diagnosis; (8) knowledge of metastatic status; (9) knowledge of surgical conditions. In total, 63,766 prostate cancer patients met these inclusion criteria, as illustrated in the detailed flow chart provided in Fig. 1.

### Ethical review

The data in the SEER database are public and patient information is hidden, so that patient ethical approval or informed consent is not required. We followed the guidelines published in the SEER database for this study. The Medical Ethics Committee of the Second Affiliated Hospital of Fujian Medical University (Approval No. IRB2023098) approved the research.

### Statistical analysis

The baseline characteristics of prostate cancer (PCa) patients were presented using descriptive statistics. Different clinicopathological factors were compared between married and unmarried groups through Pearson’s chi-square tests for various variables, with categorical variables expressed as frequencies (percentages). The endpoints of this study are overall survival (OS) and cancer-specific survival (CSS). They were analyzed using the Kaplan–Meier method and the log-rank test to compare survival differences between groups.

Independent prognostic factors and hazard ratios (HR) for OS and CSS were identified using univariate and multivariate Cox proportional hazard models. Variables with prognostic significance in the univariate analysis (*P* < 0.05) were included in the final multivariate analysis. To minimize covariate differences between groups, a 1:1 propensity score matching (PSM) analysis was conducted as follows: (a) marital status (married and unmarried) was calculated using a binary logistic regression model that included all the covariates mentioned above; (b) 1:1 matching was performed using the nearest neighbor algorithm; (c) matching between married and unmarried patients was conducted, with all baseline covariates having P-values > 0.001 based on a standardized difference (SD) of < 0.1, indicating balanced covariates between the two groups.

The Kaplan–Meier analysis, Cox proportional hazards regression, and 1:1 propensity score matching were performed using R version 4.3.1. The R packages used in this study included Table1, MatchIt, tableone, rms, survival, survminer, ezcox, and ggplot2. All P-values were two-sided, and *P* < 0.05 was considered statistically significant.

**Table 1:** Comparison of baseline characteristics between married and unmarried groups.

	Unmarried	Married	*P*-value
	(N = 15,649)	(N = 48,117)	
Race			
Black	3351 (21.4%)	4561 (9.5%)	< 0.001
Other	906 (5.8%)	3673 (7.6%)	
White	11,392 (72.8%)	39,883 (82.9%)	
Age			
< 65 years	7807 (49.9)	21,421 (44.5)	< 0.001
≥ 65 years	7842 (50.1)	26,696 (55.5)	
Year of diagnosis is			
2010	2862 (18.3)	9349 (19.4)	< 0.001
2011	2672 (17.1)	8888 (18.5)	
2012	2442 (15.6)	7547 (15.7)	
2013	2519 (16.1)	7368 (15.3)	
2014	2511 (16.0)	7214 (15.0)	
2015	2643 (16.9)	7751 (16.1)	
Grade			
Well differentiated, grade I	1461 (9.3%)	4557 (9.5%)	0.001
Moderately differentiated, grade II	5830 (37.3%)	18,577 (38.6%)	
Poorly differentiated, grade III	8333 (53.2%)	24,942 (51.8%)	
Undifferentiated, grade IV	25 (0.2%)	41 (0.1%)	
T (6th)			
T0–T1	7108 (45.4%)	18,539 (38.5%)	< 0.001
T2	6274 (40.1%)	22,468 (46.7%)	
T3	1772 (11.3%)	5989 (12.4%)	
≥ T4	495 (3.2%)	1121 (2.3%)	
N (6th)			
N0	14,453 (92.4%)	45,371 (94.3%)	< 0.001
N1	756 (4.8%)	1636 (3.4%)	
NX	440 (2.8%)	1110 (2.3%)	
M (6th)			
M0	14,443 (92.3%)	45,749 (95.1%)	< 0.001
M1	1025 (6.6%)	1863 (3.9%)	
MX	181 (1.2%)	505 (1.0%)	
Radiation			
No/unknown	9433 (60.3%)	30,058 (62.5%)	< 0.001
Yes	6216 (39.7%)	18,059 (37.5%)	
Chemotherapy			
No/unknown	15,475 (98.9%)	47,756 (99.2%)	< 0.001
Yes	174 (1.1%)	361 (0.8%)	
Surgery			
No	10,376 (66.3%)	27,027 (56.2%)	< 0.001
Yes	5273 (33.7%)	21,090 (43.8%)	
Gleason score			
GS ≤ 7	11,890 (76.0%)	38,320 (78.7%)	< 0.001
GS > 7	3759 (24.0%)	9797 (20.4%)	
PSA			
< 98	14,848 (94.9%)	47,004 (97.7%)	< 0.001
≥ 98	801 (5.1%)	1113 (2.3%)	

### Ethics approval and consent to participate

The SEER database provides publicly available data for this study, which means that obtaining informed consent from participants or ethical approval from an institutional review board is not necessary. We obtained access to the 1979–2019 SEER Research Data File by signing a Data-Use Agreement that outlines the terms and conditions for access. The Medical Ethics Committee of the Second Affiliated Hospital of Fujian Medical University (Approval No. IRB2023098) approved the research.

## Results

### Baseline demographic and clinical characteristics of PCa patients

This study involved a total of 63,766 prostate cancer (PCa) patients diagnosed between 1975 and 2019. Detailed information on their demographic and clinical characteristics is presented in Table 1. Among these patients, 48,117 (75.46%) were categorized in the married group, while 15,649 (24.54%) were in the unmarried group. This highlights that the majority of the included patients were married.

Table 1 provides insights into the data, indicating that variables such as race, age, year of diagnosis, tumor stage, TNM stage, radiotherapy, chemotherapy, surgery, Gleason score, and PSA value exhibited statistical significance (*P* < 0.05). Regarding treatment methods, the married group had nearly 10% more patients who underwent surgical resection, suggesting a potential association between receiving surgical treatment and marital status.

Furthermore, it is worth noting that the unmarried group had a nearly equal distribution of patients under and over 65 years of age at the time of diagnosis (49.9% vs. 50.1%), whereas the married group had a significantly higher proportion of patients over 65 years old (55.5% vs. 44.5%).

### COX regression analysis of the independent prognostic factors related to mortality in PCa patients

In the univariate Cox regression analysis, 12 variables were found to have significant associations with the overall survival (OS) of prostate cancer (PCa). These variables include marital status (unmarried group, HR: 1.043, 95% CI [1.022–1.065], *P* < 0.001), age (65 years, HR: 1.03, 95% CI [1.012–1.047], *P* < 0.001), race (White, HR: 0.921, 95% CI [0.898–0.946], *P* < 0.001), Grade stage (II, HR: 0.279, 95% CI [0.270–0.287], *P* = 0; III, HR: 0.22, 95% CI [0.214–0.227], *P* = 0; IV, HR: 0.18, 95% CI [0.132–0.246], *P* < 0.001), T stage (6th) (T2, HR: 0.922, 95% CI [0.905–0.939], *P* < 0.001; T3, HR: 1.06, 95% CI [1.030–1.090], *P* < 0.001; ≥ T4, HR: 1.19, 95% CI [1.120–1.280], *P* < 0.001), N stage (6th) (N2, HR: 1.31, 95% CI [1.240–1.380], *P* < 0.001; Nx, HR: 1.14, 95% CI [1.070–1.220], *P* < 0.001), M stage (6th) (M1, HR: 1.34, 95% CI [1.250–1.440], *P* < 0.001), radiotherapy (receiving radiotherapy, HR: 0.946, 95% CI [0.930–0.963]), chemotherapy (receiving chemotherapy, HR: 1.61, 95% CI [1.420–1.830]), surgery (received surgical HR: 0.96, 95% CI [0.944–0.977]), Gleason Clinical Scale (GS) (GS > 7, HR: 1.17, 95% CI [1.140–1.200]), and PSA concentration (> 98 ng/ml, HR: 1.18, 95% CI [1.080–1.300]). For cancer-specific survival (CSS), all prognostic factors were the same as for OS (*P* < 0.001), except for radiotherapy (HR: 0.991, 95% CI [0.975–1.010], *P* = 0.300).

In the multivariate Cox regression analysis, which included the univariate Cox regression analysis to further investigate survival factors, we identified marital status, age, race, Grade stage, TNM (6th) stage, radiotherapy, chemotherapy, surgery, and Gleason score > 7 as independent prognostic factors significantly affecting prostate cancer (Table 2).

**Table 2:** Univariate and multivariate analysis of the in PCa.

Variables	OS				CSS			
	Univariate analysis		Multivariate analysis		Univariate analysis		Multivariate analysis	
	HR (95% CI)	*P* -value	HR (95% CI)	*P* -value	HR (95% CI)	*P* -value	HR (95% CI)	*P* -value
Marital status								
Married	Reference		Reference		Reference		Reference	
Unmarried	1.043 (1.022–1.065)	< 0.001	1.016 (0.995–1.037)	0.141	1.100 (1.080–1.130)	< 0.001	1.098 (1.078–1.120)	< 0.001
Age								
< 65 years	Reference							
≥ 65 years	1.030 (1.012–1.047)	< 0.001	0.967 (0.950–0.984)	< 0.001	1.160 (1.140–1.180)	< 0.001	1.173 (1.154–1.193)	< 0.001
Race								
Black	Reference				Reference			
White	0.921 (0.898–0.946)	< 0.001	0.973 (0.947–0.999)	0.046	0.918 (0.896–0.941)	< 0.001	0.886 (0.864–0.908)	< 0.001
Other (American Indian/AK Native, Asian/Pacific Islander)	0.971 (0.933–1.01)	0.146	1.020 (0.980–1.062)	0.336	0.964 (0.929–1.000)	0.0563	0.886 (0.864–0.908)	< 0.001
Grade								
Well differentiated, grade I	Reference		Reference		Reference		Reference	
Moderately differentiated, grade II	0.279 (0.270–0.287)	0	1.002 (0.970–1.035)	0.925	0.329 (0.319–0.339)	0	0.319 (0.310–0.329)	0
Poorly differentiated, grade III	0.220 (0.214–0.227)	0	1.015 (0.977–1.055)	0.442	0.279 (0.271–0.287)	0	0.223 (0.216–0.231)	0
Undifferentiated, grade IV	0.180 (0.132–0.246)	< 0.001	1.019 (0.746–1.394)	0.904	0.227 (0.170–0.304)	< 0.001	0.156 (0.117–0.209)	< 0.001
T stage (6th)								
T0–T1	Reference		Reference		Reference		Reference	
T 2	0.922 (0.905–0.939)	< 0.001	0.972 (0.949–0.994)	0.015	0.891 (0.876–0.906)	< 0.001	1.053 (1.031–1.076)	< 0.001
T 3	1.060 (1.030–1.090)	< 0.001	0.984 (0.950–1.019)	0.36	0.996 (0.970–1.020)	0.789	1.219 (1.179–1.260)	< 0.001
≥ T4	1.190 (1.120–1.280)	< 0.001	0.994 (0.926–1.066)	0.856	1.180 (1.110–1.260)	< 0.001	1.253 (1.173–1.337)	< 0.001
N stage (6th)								
N0	Reference		Reference		Reference		Reference	
N1	1.310 (1.240–1.380)	< 0.001	1.010 (0.954–1.069)	0.741	1.280 (1.220–1.350)	< 0.001	1.102 (1.045–1.163)	< 0.001
Nx	1.140 (1.070–1.220)	< 0.001	1.056 (0.974–1.145)	0.188	1.240 (1.170–1.310)	< 0.001	1.102 (1.025–1.185)	0.009
M stage (6th)								
M0	Reference		Reference		Reference		Reference	
M1	1.340 (1.250–1.440)	< 0.001	1.068 (0.983–1.160)	0.118	1.640 (1.550–1.740)	< 0.001	1.350 (1.259–1.447)	< 0.001
Mx	1.080 (0.985–1.170)	0.106	0.976 (0.873–1.091)	0.666	1.150 (1.060–1.250)	< 0.001	0.930 (0.841–1.028)	0.156
Radiation								
No/unknown	Reference		Reference		Reference		Reference	
Yes	0.946 (0.930–0.963)	< 0.001	0.945 (0.925–0.967)	< 0.001	0.991 (0.975–1.010)	0.300	1.004 (0.984–1.025)	0.703
Chemotherapy								
No/unknown	Reference		Reference		Reference		Reference	
Yes	1.610 (1.420–1.830)	< 0.001	0.957 (0.836–1.095)	0.519	1.550 (1.380–1.750)	< 0.001	1.235 (1.092–1.396)	< 0.001
Surgery								
No	Reference		Reference		Reference		Reference	
Yes	0.960 (0.944–0.977)	< 0.001	0.979 (0.953–1.005)	0.111	0.879 (0.865–0.894)	< 0.001	0.999 (0.974–1.023)	0.909
Gleason score								
GS ≤ 7	Reference		Reference		Reference		Reference	
GS > 7	1.170 (1.140–1.200)	< 0.001	1.020 (0.992–1.049)	0.167	1.280 (1.250–1.310)	< 0.001	1.539 (1.502–1.578)	< 0.001
PSA								
PSA < 98	Reference		Reference		Reference		Reference	
PSA ≥ 98	1.180 (1.080–1.300)	< 0.001	0.946 (0.867–1.033)	0.216	1.480 (1.380–1.580)	< 0.001	1.105 (1.025–1.191)	0.009

### For propensity score matching in prostate cancer patients

In order to mitigate potential confounding variables between the married and unmarried groups, including factors such as age, race, Grade stage, TNM stage (6th edition), radiotherapy, chemotherapy, surgery, Gleason score, and PSA levels, we employed a 1:1 propensity score matching method. This matching process resulted in 15,258 married patients and an equal number of unmarried patients (Table 3). Importantly, the baseline matching between the two groups was robust, with no statistically significant differences observed (*P* > 0.05).

**Table 3:** Baseline characteristics between married and unmarried groups after propensity score matching.

Characteristic	Unmarried (N = 15,258)	Married (N = 15,258)	*P* -value
Age			
< 65 years	7586 (49.7)	7536 (49.4)	0.575
≥ 65 years	7672 (50.3)	7722 (50.6)	
Race			
Black	3109 (20.4)	3008 (19.7)	0.26
White	11,283 (73.9)	11,408 (74.8)	
Other (American Indian/AK Native, Asian/Pacific Islander)	866 (5.7)	842 (5.5)	
Grade			
Well differentiated, grade I	1448 (9.5)	1440 (9.4)	0.994
Moderately differentiated, grade II	5763 (37.8)	5760 (37.8)	
Poorly differentiated, grade III	8037 (52.7)	8047 (52.7)	
Undifferentiated, grade IV	10 (0.1)	11 (0.1)	
T stage (6th)			
T0–T1	6985 (45.8)	7008 (45.9)	0.416
T2	6169 (40.4)	6058 (39.7)	
T3	1691 (11.1)	1761 (11.5)	
T4	413 (2.7)	431 (2.8)	
N stage (6th)			
N0	14,256 (93.4)	14,177 (92.9)	0.128
N1	642 (4.2)	714 (4.7)	
Nx	360 (2.4)	367 (2.4)	
M stage (6th)			
M0	14,289 (93.6)	14,158 (92.8)	0.007
M1	820 (5.4)	949 (6.2)	
Mx	149 (1.0)	151 (1.0)	
Chemotherapy			
No/unknown	15,142 (99.2)	15,135 (99.2)	0.697
Yes	116 (0.8)	123 (0.8)	
Surgery			
No	10,062 (65.9)	10,123 (66.3)	0.468
Yes	5196 (34.1)	5135 (33.7)	
Gleason score			
GS > 7	3508 (23.0)	3622 (23.7)	0.126
GS ≤ 7	11,750 (77.0)	11,636 (76.3)	
PSA			
PSA < 98	14,666 (96.1)	14,625 (95.9)	0.243
PSA ≥ 98	592 (3.9)	633 (4.1)	

### Single multivariate COX regression analysis and Kaplan–Meier survival analysis were performed again

**Figure 2:**
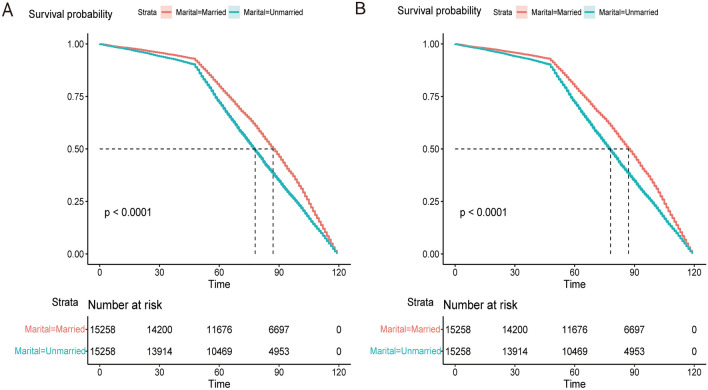
The Kaplan–Merier curve for marriage and survival status after propensity score matching. (**A**) OS curve; (**B**) CSS curve.

After performing 1:1 propensity score matching, we obtained two groups: the married group (n = 15,258) and the unmarried group (n = 15,258), resulting in a total of 30,516 prostate cancer (PCa) patients (Table 4). When examining marital status, men in the unmarried group exhibited a significantly higher hazard ratio (HR) of 1.61 (95% CI 1.34–1.93) for overall survival (OS) and cancer-specific survival (CSS) when adjusting for covariates including age, race, Grade stage, TNM stage (6th edition), chemotherapy, surgery, Gleason score, and PSA levels, as compared to men in the married group (*P* < 0.001) (Fig. 2).

**Table 4:** Single multivariate COX regression analysis of marital status and prostate cancer outcomes after propensity score matching.

Variables	Univariate analysis			
	OS		CSS	
	HR (95% CI)	P-value	HR (95% CI)	P-value
Marital status				
Married (15,258)	Reference		Reference	
Unmarried (15,258)	0.835 (0.814–0.856)	< 0.001	0.805 (0.786–0.824)	< 0.001

### Effect of marital status on overall survival (OS) and cancer-specific survival (CSS) in patients with PCa

**Figure 3:**
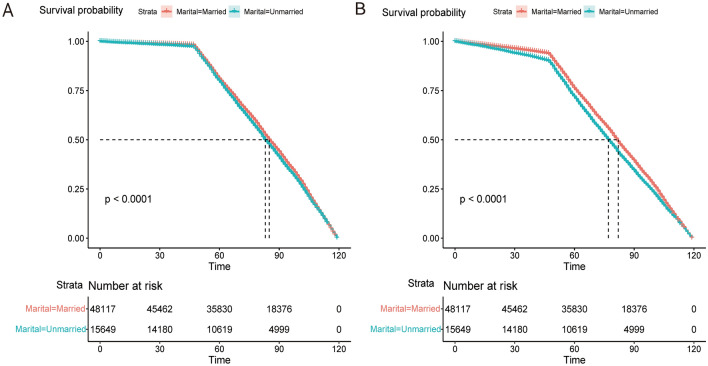
The Kaplan–Merier curve for marriage and survival status before propensity score matching. (**A**) OS curve; (**B**) CSS curve.

We conducted a Kaplan–Meier analysis to investigate the impact of marital status on both overall survival (OS) and cancer-specific survival (CSS). The results showed that the 5-year OS rate and CSS rate for married patients were significantly higher at 75.5% compared to the 24.5% observed in unmarried patients (*P* < 0.001) (Fig. 3).

### Subgroup analysis of the effects of different marital status on OS and CSS in PCa patients

Our research has revealed a significant influence of marital status on the survival of prostate cancer patients. Furthermore, our analysis, based on a multivariable Cox proportional hazards regression model, identified several variables, including age, Gleason score, and PSA concentration, as critical risk factors for mortality in patients with prostate cancer (PCa). Consequently, we conducted an in-depth examination of the impact of different marital statuses (divorced/separated: 5742, married: 48,117, single/unmarried: 7512, widowed: 2395) on overall survival (OS) and cancer-specific survival (CSS) within various subgroups of PCa patients.

**Figure 4:**
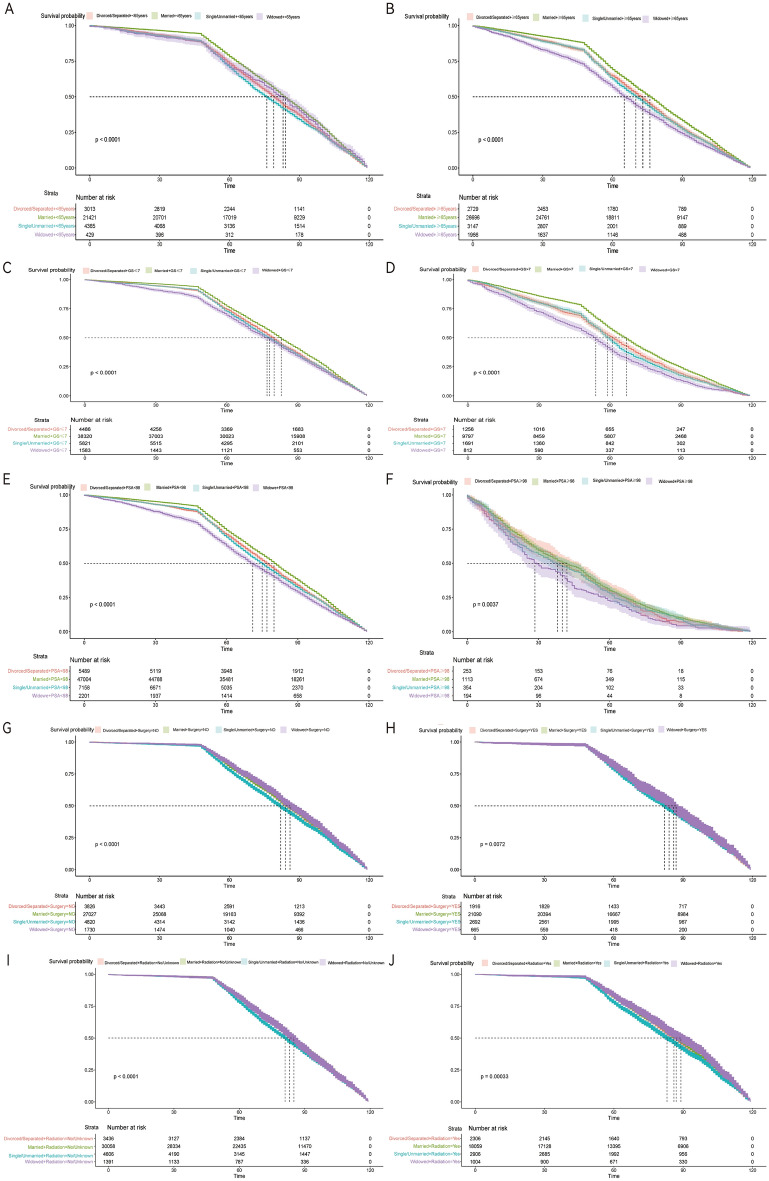
Kaplan–Meier survival curves in patients with PCa of different age/Gleason score/PSA. (**A**,**B**) Age and marital; (**C**,**D**) Gleason score and marital; (**E**,**F**) PSA and marital; (**G**,**H**) surgery and marital; (**I**,**J**) radiation and marital.

We stratified a total of 63,766 patients based on established variables, including age (< 65 years and ≥ 65 years), Gleason grade (GS < 7 and GS ≥ 7), radiation (yes and no/unknown), surgery (Yes and No) and PSA concentration (PSA < 98 ng/ml and PSA ≥ 98 ng/ml). Our results consistently demonstrated that patients who were married exhibited the highest overall survival rates, irrespective of age (see Fig. 4A,B), as well as within Gleason score-stratified subgroups (refer to Fig. 4C,D). Moreover, when we stratified these patients based on PSA concentration, married patients continued to display a significant survival advantage, with the risk of death from overall survival (OS) remaining consistently highest across all comparisons (refer to Fig. 4E,F). These findings collectively indicate that marital status remains an independent prognostic factor for enhancing overall survival in the majority of subgroups. However, for the treatment modalities, such as radiotherapy and surgery, the survival status is different (As shown in Fig. 4G–J). Among all the untreated patients, the highest (*P* < 0.001) survival, and among the patients treated with and without radiotherapy, marital status had statistically different groups, with the highest survival for the widowed patients (*P* < 0.001).

## Discussion

### The impact of marital status on prostate cancer patients stratified by age and other key variables

This study aimed to stratify the independent risk factors for prostate cancer and investigate the influence of marital status on the survival of prostate cancer (PCa) patients. We initially processed data from 584,655 cases obtained from the SEER database, resulting in a final dataset of 63,766 cases. Following univariable and multivariable regression analyses, marital status emerged as an independent prognostic factor for both overall survival (OS) and cancer-specific survival (CSS). Particularly, a subgroup analysis, based on the independent prognostic factors identified in this study, revealed that marital status independently predicts mortality risk in subgroups characterized by varying ages, Gleason scores, whether surgery and radiation therapy treatment and PSA concentrations. To mitigate the impact of confounding variables between married and unmarried groups, we conducted a 1:1 propensity score matching analysis, which involved matching 15,258 controls. The analysis reaffirmed that marital status remains an independent prognostic and protective factor for PCa patients. In the subgroup analysis of marital status within this experiment, we observed that the highest OS among widowed patients with PSA concentrations < 98 ng/ml or not receiving surgery or radiotherapy was largely associated with the smallest number of widowed patients in the overall cohort (5-year OS: 1414/2395; 1040/2395; 787/2395). Psychosocial factors may explain some of the disparities in OS. For instance, married men may be more inclined to opt for treatments with fewer side effects. Alternatively, previous research has indicated that widowed seniors tend to exchange more daily support with friends or relatives compared to their married counterparts, possibly due to shifts in family responsibilities^11^ and related with stress reduction such as spouse caring^12^.

### The impact of marital status on survival in cancer and other chronic diseases

In recent years, numerous studies have underscored the crucial role of social support in influencing the survival rates of cancer patients. For instance, Leif reported that patients with medullary thyroid carcinoma (MTC) who were married exhibited a more favorable prognosis compared to their unmarried counterparts^13^. Similarly, Chen identified marital status as an independent prognostic factor for cancer-specific survival among patients with non-small cell lung cancer (NSCLC)^14^. Furthermore, a comprehensive meta-analysis and review, encompassing 63 relevant articles, highlighted that married individuals had higher overall survival and cancer-specific survival rates in comparison to unmarried individuals, which includes singles, those who are unmarried, divorced/separated, and widowed patients^10^. However, these studies have often overlooked the influence of confounding factors. To enhance the comparability among patients with different marital statuses, we conducted 1:1 propensity matching using the surveillance, epidemiology, and end results (SEER) database. This approach mitigated the impact of confounding variables and allowed us to identify eligible prostate cancer patients, yielding more reliable results based on well-matched data. Marital status, age, race, grade stage, TNM stage, radiotherapy, chemotherapy, surgery, Gleason score ≤ 7, and PSA concentration ≥ 98 ng/ml emerged as independent prognostic factors significantly affecting prostate cancer. Married patients largely experienced better survival outcomes, while single/unmarried patients faced poorer overall survival and cancer-specific survival rates.

### The significance of marital status in prostate cancer patients

Our current research highlights the pivotal role that marital status plays in the context of prostate cancer. Cancer, as a formidable stressor for patients, underscores the importance of emotional support, particularly from a spouse, in alleviating the adverse effects of stress. This, in turn, can lead to improved treatment outcomes. We posit several possible explanations for this phenomenon: Firstly, the presence of a partner or spouse may lead to beneficial neurological and physiological changes. Research has demonstrated that the activation of neural regions associated with threat is correlated with elevated cortisol levels and a heightened inflammatory response to stressors^15^. Additionally, there is evidence suggesting that emotional support from romantic partners prior to stressful tasks can diminish the activation of neural regions associated with fear and reduce cortisol responses to social stress. Intimate expressions of physical bonding have also been associated with lower daily cortisol levels, especially among individuals experiencing high levels of job-related stress^16^. Likewise, holding a romantic partner's hand has been shown to attenuate neural activation in brain regions associated with threat and emotion regulation, while the mere presence of a caring partner reduces the need for individuals to mobilize their personal resources when addressing environmental challenges^17^. Furthermore, a loving and caring partner is linked to an increased release of oxytocin, which can inhibit the growth of cancer cells through both indirect and direct mechanisms^13^. Secondly, married patients are more likely to detect the disease at an earlier stage and opt for active treatment. Studies have consistently shown that married patients, including those with non-Hodgkin’s lymphoma, are more likely to receive chemotherapy and experience higher survival rates compared to their unmarried counterparts. This is in line with findings across various other cancer types. The reasons behind this trend may include the involvement and support of family members in the treatment plan and a perceived higher ‘value’ of life^18^. Thirdly, married patients tend to enjoy better mental health. Optimism has been significantly linked to reduced anxiety and depressive symptoms, decreased feelings of despair, and an enhanced overall quality of life. Social support is similarly associated with improved quality of life, and both optimism and social support contribute to enhanced overall well-being among cancer patients. These factors are likely to contribute to improved survival outcomes for prostate cancer patients and underscore the significance of one’s mindset in facing end-of-life challenges^19^. Lastly, married patients generally have a higher household income. Economic circumstances can influence a patient's adherence to treatment and choice of medication, among other factors. A more favorable financial situation can support patients in undergoing systematic and standardized treatment regimens, ultimately leading to improved quality of life and survival outcomes.

### Limitations

Firstly, it’s important to note that the SEER database lacks detailed information regarding the duration and quality of marriages, as well as the history of marital relationships. This limitation hinders our ability to conduct a comprehensive assessment. While our study has demonstrated improved survival outcomes for married prostate cancer patients, it’s worth acknowledging that certain studies have reported that marital distress is associated with a slower trajectory of cancer recovery and poorer outcomes. Specifically, individuals in troubled relationships have consistently exhibited higher overall stress levels, deteriorating health behaviors, slower improvements in cancer-specific stress, and performance status, ultimately resulting in higher functional impairment when compared to those in healthier relationships^20^. Hence, there is a need for further research to explore the impact of marital satisfaction and marital quality on cancer outcomes, with the aim of enhancing outcomes for cancer patients.

Secondly, the SEER database records only legally recognized marriages, potentially leading to an underestimation of survival outcomes for individuals in non-traditional relationships, such as gay and transgender individuals. Thirdly, the SEER database considers marital status at the time of diagnosis as a fixed and unchangeable attribute. However, patients may experience changes in marital status throughout the course of their lives and treatments. Such changes in marital status may be accompanied by alterations in economic circumstances, marital quality, and the availability of social support. Factors that could not be included in our study may introduce bias into the results. Fourthly, it’s worth noting that the SEER database primarily consists of white and black populations, which could limit the generalizability of our study’s findings to other ethnic groups, such as individuals of Asian descent. Therefore, further and more comprehensive epidemiological investigations among diverse ethnic populations are warranted to improve the applicability of our study’s results. At the same time, the variable PSA selected in this study uses a threshold of 98 ng/ml. However, only 5% of patients have PSA levels greater than 98 ng/ml, which is far from the clinical diagnosis and treatment threshold. Therefore, we hope to make further attempts in the future to determine a more appropriate clinical threshold. Finally, previous studies have indicated that factors like socioeconomic status^21^, social cognition, emotional processes^22^, and lifestyle choices^23^ could potentially act as confounding variables in the relationship between marital status and the prognosis of prostate cancer patients. These factors may still have a significant impact, but our study did not adequately balance these variables. Therefore, incorporating balanced variables into the propensity score matching is necessary to minimize the influence of confounding factors.

## Conclusion

Based on our analysis of previous research findings and our own expectations, we have determined that marital status serves as an independent prognostic and protective factor for the survival of prostate cancer (PCa) patients. Specifically, married patients tend to experience better outcomes. Utilizing the extensive SEER database, we have identified several crucial predictive risk factors for PCa, including age, TNM stage, Gleason score, and PSA concentration. Notably, these factors align with the criteria used by the national comprehensive cancer network (NCCN) to define high-risk prostate cancer. Therefore, this study holds significant potential for aiding in the early detection and treatment of high-risk prostate cancer. Its implications extend beyond just extending the patient’s life; it can also have a positive impact on the patient’s family by reducing the family’s care-giving burden. These findings carry important clinical implications.

## Data Availability

The data used in this study are available free of charge online at http://www.seer.cancer.gov on request.
